# Feasibility trial of an early therapy in perinatal stroke (eTIPS)

**DOI:** 10.1186/s12883-018-1106-4

**Published:** 2018-07-23

**Authors:** Anna Purna Basu, Janice Pearse, Rose Watson, Pat Dulson, Jessica Baggaley, Blythe Wright, Denise Howel, Luke Vale, Dipayan Mitra, Nick Embleton, Tim Rapley

**Affiliations:** 1 Institute of Neuroscience, Newcastle University, Newcastle upon Tyne, NE1 7RU UK; 2 Department of Paediatric Neurology, Newcastle upon Tyne Hospitals NHS Foundation Trust, Newcastle upon Tyne, NE7 7DN UK; 3 Therapy Services, Newcastle upon Tyne Hospitals NHS Foundation Trust, Newcastle upon Tyne, NE7 7DN UK; 4 Institute of Health and Society, Newcastle University, Newcastle upon Tyne, NE2 4AX UK; 5 Newcastle Neonatal Service, Newcastle upon Tyne Hospitals NHS Foundation Trust, Newcastle upon Tyne, UK; 6 Human Biosciences, Northumbria University, Newcastle upon Tyne, NE1 8ST UK; 7 Department of Neuroradiology, Newcastle upon Tyne Hospitals NHS Foundation Trust, Newcastle upon Tyne, NE7 7DN UK; 8 Department of Social Work, Education and Community Wellbeing, Northumbria University, Coach Lane Campus West, Newcastle upon Tyne, NE7 7XA UK

**Keywords:** Early intervention, Therapy, Hand function, Infant, Perinatal stroke, Haemorrhagic parenchymal infarction, Parent-delivered therapy, Feasibility trial

## Abstract

**Background:**

Perinatal stroke (PS) affects up to 1/2300 infants and frequently leads to unilateral cerebral palsy (UCP). Preterm-born infants affected by unilateral haemorrhagic parenchymal infarction (HPI) are also at risk of UCP. To date no standardised early therapy approach exists, yet early intervention could be highly effective, by positively influencing processes of activity-dependent plasticity within the developing nervous system including the corticospinal tract. Our aim was to test feasibility and acceptability of an “early Therapy In Perinatal Stroke” (eTIPS) intervention, aiming ultimately to improve motor outcome.

**Methods:**

Design: Feasibility trial, North-East England, August 2015–September 2017. Participants were infants with PS or HPI, their carers and therapists. The intervention consisted of a parent-delivered lateralised therapy approach starting from term equivalent age and continuing until 6 months corrected age. The outcome measures were feasibility (recruitment and retention rates) and acceptability of the intervention (parental questionnaires including the Warwick-Edinburgh Mental Wellbeing Scale (WEBWMS), qualitative observations and in-depth interviews with parents and therapists). We also reviewed clinical imaging data and undertook assessments of motor function, including the Hand Assessment for Infants (HAI). Assessments were also piloted in typically developing (TD) infants, to provide further information on their ease of use and acceptability.

**Results:**

Over a period of 18 months we screened 20 infants referred as PS/HPI: 14 met the inclusion criteria and 13 took part. At 6 months, 11 (85%) of those enrolled had completed the final assessment. Parents valued the intervention and found it acceptable and workable. There were no adverse events related to the intervention. We recruited 14 TD infants, one of whom died prior to undertaking any assessments and one of whom was subsequently found to have a condition affecting neurodevelopmental progress: thus, data for 12 TD infants was analysed to 6 months. The HAI was well tolerated by infants and highly valued by parents. Completion rates for the WEBWMS were high and did not suggest any adverse effect of engagement in eTIPS on parental mental wellbeing.

**Conclusion:**

The eTIPS intervention was feasible to deliver and acceptable to families. We plan to investigate efficacy in a multicentre randomised controlled trial.

**Trial registration:**

ISRCTN12547427 (registration request submitted 28/05/2015; retrospectively registered, 30/09/2015).

## Background

Perinatal stroke (PS) is due to an interrupted blood supply to part of the brain before birth or in the first 28 days of life [1]. Perinatal arterial ischaemic stroke affects around 1/2300 term [1, 2] and 7/1000 preterm deliveries [1, 3]. Some infants present with seizures and encephalopathy, whilst others (around 40%) appear asymptomatic in the neonatal period though signs of unilateral cerebral palsy (UCP) emerge over time. Furthermore, not all infants who sustain a perinatal stroke will have an abnormal motor outcome, though up to 60% do have neurological deficits [4]: the risk of developing UCP can be assessed through cranial imaging [5]. Perinatal stroke remains one of the leading causes of UCP, with associated lifelong morbidity affecting function in activities of daily living [6].

Preterm infants with unilateral haemorrhagic parenchymal infarcts (HPI) after grade IV intraventricular haemorrhage (IVH) are also at high risk of developing UCP. In a study from 2006, HPI was observed in 1% of all premature infants with a birthweight of under 2500 g, occurring more frequently in infants with lower birthweight and lower gestational age [7]. 74% of cases of HPI are unilateral [8, 9]. The pathophysiology of HPI differs from that of arterial ischaemic stroke – it is a form of venous infarction due to impaired drainage from veins in the periventricular white matter because of pressure from the intraventricular haemorrhage [10]. In a study by Maitre et al., [9] 67% of patients with unilateral HPI developed cerebral palsy, with UCP being the commonest form. Imaging (including cranial ultrasound) provides some guidance regarding the risk of developing UCP [11], though tractography within the first 4 weeks of life and MRI at term equivalent age may be more accurate [12].

Whilst the pattern of neuronal damage and the nature and scope for reorganisation differ between these two forms of injury [13], both frequently lead to UCP, and for both conditions, options for primary prevention are limited [14, 15]. For symptomatic cases, stem cell therapy and neuroprotection are under investigation as part of acute management [16], but there remains no established approach except symptomatic management. Therapy intervention programmes aiming to improve hand function exist for infants and children with established UCP [17], with evidence of benefit from high-dose constraint-induced movement therapy and bimanual therapy [18]. However, there has been little focus on early therapy intervention in the period between onset of the brain insult and emergence of UCP. This is despite extensive evidence from studies demonstrating ongoing activity-dependent corticospinal tract plasticity [19, 20] which could be modulated during this early time window with the potential for a greater influence on motor outcomes than with later interventions [16]. We have detailed elsewhere the rationale for an early lateralised therapy approach [21].

Prior to undertaking this feasibility trial, we confirmed the lack of a recognised evidence-based alternative early therapy approach to perinatal stroke through a national (UK-based) survey of current practice [22]. Interventions such as early modified constraint-induced movement therapy (“Baby CIMT”), and early intensive bimanual task-specific training, are under investigation outside the UK but do not have definitive evidence of effectiveness to date [23, 24]. In conjunction with key stakeholders, we developed a novel parent-delivered pervasive therapy approach aiming to promote activity of the potentially affected side of the body from as soon as possible after diagnosis. Details of the intervention and the development process have already been published [21]. The aims of this trial were to assess feasibility of the intervention and to pilot the outcome assessments prior to proceeding to a definitive randomised controlled trial.

### Objectives

Our primary objectives were:

1. To establish feasibility of delivery and the acceptability of an early parent-delivered home-based therapy intervention in PS/HPI and identify and address potential barriers to implementation.
2. To obtain information on rates of eligibility, consent, participation and retention.
3. To pilot assessments and outcome measures for use in a future trial.

## Methods

### Trial design

We conducted a feasibility trial of the eTIPS intervention in infants with PS/HPI. All infants with PS/HPI received the intervention, to maximise our experience with delivery at the feasibility stage. We recruited an equal number of typically developing (TD) infants to undertake the assessments, but TD infants did not undertake the eTIPS intervention: the inclusion of TD infants gave us additional information on the ease of use and acceptability of the assessments.

### Eligibility criteria for participants

Eligible infants were recruited from four hospitals with level three neonatal units; a further four other hospitals were added as participant identification centres to avoid missing potentially eligible infants. Inclusion criteria were: a) term or preterm infants who sustained a predominantly unilateral stroke (arterial ischaemic, haemorrhagic or haemorrhagic periventricular venous infarction) demonstrated on cranial imaging and identified within the first 3 months of life, b) fully informed parental consent and c) ability and willingness of the parent/carer to adhere to the protocol. Participants were not eligible if they had a) additional significant medical diagnoses which would render the therapy inappropriate or outcomes uninterpretable in relation to the therapy, e.g. known progressive or neurodegenerative disorder or severe visual impairment, b) evidence of significant bilateral intracerebral motor pathology, c) strokes shown radiologically to affect only occipital, prefrontal or temporal areas of the brain (which would not be expected to produce adverse motor outcomes), or d) ongoing involvement in another research study where this was likely to interfere with the interpretation of either study. Initially we had a further exclusion criterion of extreme prematurity (less than 26 weeks gestation), but after discussions with neonatologists in the first 3 months of the study we decided that cases should be considered regardless of gestation, to avoid missing otherwise eligible recruits, and because the incidence of HPI is higher in infants with lower gestational age. Thirteen TD infants (including preterm infants with gestational ages matching those of the infants with HPI) were also recruited to obtain comparative data for exploratory assessments of limb movements, and to pilot the infant massage materials we developed as a potential attentional control, as described below. All TD infants were recruited from the Newcastle site: preterm TD infants were recruited from the neonatal unit through the same procedures as for the infants with PS/HPI, whilst term infants were recruited through postnatal wards and through provision of flyers approved by the ethics committee.

### Participant identification and consent procedure

Between August 2015 and January 2017, clinical staff at participating centres and sites identified and approached parents/carers of potential infant participants, providing flyers and information sheets. These materials were developed with the involvement of a parent of a young child with UCP. With parental consent, contact details were forwarded to a member of the eTIPS team, and they were then screened for eligibility to participate in the trial. If eligible, written informed consent was obtained. Parents/carers of infants in the trial were also recruited as participants, so we could capture their experiences regarding the therapy and assessments. We included mothers, fathers and grandparents if actively involved in the infant’s care on a regular basis, and allowed more than one such carer to participate per infant. After commencing the trial, we also sought permission to recruit (with parental permission) therapists involved in the clinical care of recruited infants, to capture their views on the approach.

### eTIPS intervention

The eTIPS intervention was developed with input from parents of children with UCP and healthcare professionals caring for these children; it is described in our intervention development paper [21]. In summary, it is a parent-delivered, pervasive, lateralised therapy intervention in the first 6 months of life, aiming to improve infant motor outcome. The therapy is incorporated into all day-to-day infant activities (infant holding, feeding, bathing, play) to promote opportunities for active use and stimulation (including the use of massage) of the potentially affected side of the body by adapting the way these activities are undertaken rather than by introducing specific blocks of therapy time into the day. The environment around the infant is also adapted to maximise opportunities to see, reach and grasp for objects on the potentially affected side.

All infants with PS/HPI received the eTIPS intervention in addition to usual National Health Service care. The intervention began when medically stable but not before term-equivalent age, and continued until 6 months of age (or for preterm infants, 6 months corrected age). At the baseline visit, parents were given education and materials covering all aspects of the eTIPS approach, including the rationale for the approach. The materials comprised a pictorial manual tailored to the side of the stroke, a DVD with videos demonstrating the desired behaviours, and password protected access to a website hosting the same materials. The manual included an introductory section (“Why have I been given this manual?”) with an overview of the approach. Subsequent sections (e.g. Day to Day Care, and Play) provided examples for parents of how to promote opportunities for, and encourage, active pre-reaching or reaching and grasping on the potentially affected side during everyday activities. Much of this consisted of very straightforward suggestions e.g. presenting suitable toys to the potentially affected side during play sessions. The manual (and parent education) also included some information on the developmental context, and on parent-infant interaction (for example, advice regarding reading and responding to infant cues).

Monthly visits (usually at home, occasionally in hospital depending on circumstances), interim telephone calls (at least monthly) and fortnightly texts from the eTIPS team provided ongoing opportunities to reinforce messages regarding the intervention, troubleshoot and support families including provision of positive feedback and encouragement. During these visits, assessments were also undertaken as described below.

### Materials provided to parents of TD infants

Parents of TD infants were also provided with a manual, and videos providing guidance on a baby massage program, accessible through a website with password protected access. The materials were developed as a possible attentional control for use in a future trial. Baby massage has been successfully used in this context in a previous trial [24].

### Assessments

Table 1 shows the schedule of assessments for infants with PS/HPI and TD infants. TD infants underwent the same assessments as infants with PS/HPI, except for the Pediatric Stroke Outcome Measure, eTIPS feasibility questionnaire, and questionnaires/interviews with therapists.

**Table 1:** Schedule of assessments

	BASELINE	1 M	2 M	3 M	4 M	5 M	6 M
Review imaging	x						
PSOM	**x**			**x**			**x**
AIMS	x		x		x		x
GMs	x	x	x	x			
HAI				x	x	x	x
Accelerometry (during GMs/HAI)	x	x	x	x	x	x	x
Qualitative observations		x			x	x	
In-depth interviews						x	
PSOC		x					x
WEBWMS		x					x
eTIPS Feasibility Questionnaire		**x**					**x**
Questionnaire for therapists		**x**					
Telephone interview with therapists						**x**	

Those in bold were undertaken for infants with PS/HPI only

*Feasibility and acceptability of the eTIPS approach* were assessed through qualitative analysis of in-depth interviews undertaken in the last month of the intervention, as well as from researcher observations recorded after undertaking visits. Acceptability and feasibility of the intervention to the families involved were the key requirement for progression to a subsequent randomised trial. Interviews with carers of TD infants focused on feasibility and acceptability of trial procedures, including experiences with baby massage and assessments. We obtained feedback from therapists involved in the clinical care of infants recruited to the eTIPS study, through a questionnaire about their practice and an in-depth interview.

Just after trial commencement we requested approval to include an eTIPS feasibility questionnaire to be completed 1 month after initiating the intervention and at the final visit by parents of infants with PS/HPI. The questionnaire was adapted from feasibility questionnaires used by Ferre et al. [25] and Wallen et al. [26]; its design was informed by Normalisation Process Theory [27]. The questionnaire had two parts. Part A contained 8 questions (answered using a 5-point Likert scale) regarding how easy or difficult the participant found the eTIPS approach. Part B had 3 questions, each answered on a continuous rating scale from 0 to 10, regarding the extent to which the approach became a familiar, normal part of the daily routine.

We also added two short questionnaires for completion by both parents 1 month after entry into the study and at the final visit, which would capture any effects on parental coping and wellbeing. The Parenting Sense of Competence scale (PSOC) and the Warwick-Edinburgh Mental Well-being Scale (WEMWBS) are fully validated and have excellent psychometric properties. The PSOC is a quick (5 min) 16-point questionnaire using a 6 point Likert scale to examine parental confidence and satisfaction with parenting, which has been studied in parents of healthy infants and children throughout the age range 0–18 years and has adequate psychometric properties [28–30]. The WEMWBS scale is validated for the measurement of mental wellbeing [31]. It is short (14 items each on a 1–5 Likert scale), quick to score (under 5 min), and contains statements phrased positively. Participants complete the scale based on their thoughts and feelings over the previous 2 weeks. Whilst there is no cut-off for low levels of mental wellbeing, mean scores in a study in Scotland were 50.7 (95% CI 50.3–51.1) [31].

#### Hand assessment for infants (HAI)

The HAI [32] is an assessment of the quality of goal-directed unimanual and bimanual actions in infants age 3–12 months with unilateral CP. A validation paper has been published [33] and the assessment is being actively used in current research [24, 34, 35]. It comprises a 10 to 15-min semi-structured play session which is video recorded. The assessment is then formally scored on 17 items each with a three-point rating scale based on the manual abilities of each hand separately (12 items; raw score range 0–24 for each hand) and bimanual hand use (5 items). The final “Both Hands” score is expressed on a scale from 0 to 100 units, with higher scores representing better hand function: it has been validated by a Rasch-model analysis. An asymmetry score is also generated. In our study, the HAI was undertaken at 3, 4, 5 and 6 months.

#### Paediatric stroke outcome measure (PSOM)

Clinical assessments at baseline, 3 and 6 m were undertaken with the PSOM [36] - the only disease-specific measure of neurological outcome after paediatric stroke [37]. The PSOM is valid, reliable and completed in around 15 min [36].

#### General movements assessments (GM)

This is a Gestalt classification of the quality of spontaneous infant movements whilst in a quiet, alert state and supine, scored from a 3–5-min-long video recording. In high-risk infants the test has a high sensitivity and specificity for prediction of cerebral palsy [38]. GM assessments were undertaken monthly until age 4 months.

#### Alberta infant motor scale (AIMS)

This 58-item test, taking under 5 min to complete, is validated for the assessment of motor performance of infants from birth to 18 months; scores can be compared against the trajectory for typically developing term and preterm infants [39–41]. The assessment was performed at birth, 2, 4 and 6 months.

#### Accelerometry

Lightweight (7 g) 3-axis wireless accelerometers (WAX9, Axivity, Newcastle upon Tyne, UK) were secured at the wrists and ankles of the infants during the HAI and GM assessments using soft straps, to obtain exploratory data. We established, through examining video footage of assessments with and without accelerometers in situ, that infant limb movements were not qualitatively affected by this procedure. In addition to the formal GM assessments, accelerometry data synchronised to video data of infant movements in supine was collected at each visit up to and including 6 months. Results of the accelerometry analysis will be reported separately.

#### Piloting of healthcare resource use data collection forms

Data collection forms on healthcare resource use, modelled on the UK working party cost questionnaire [42] were piloted in all families in the study at the 3 and 6 month visits.

### Rationale for sample size

As this was a feasibility study, a sample size calculation was not performed [43]. The sample size was chosen pragmatically based on the expected number of cases in the recruitment area within the pre-specified recruitment period. A sample size of 12 affected infants, supplemented by interviews with a similar number of parents of TD infants, was expected to be adequate to reach data saturation regarding the emergence of themes from the qualitative interviews [44].

### Data analysis

Qualitative data analysis was theoretically informed by Normalisation Process Theory [27]. This provides a framework upon which to consider factors influencing the incorporation into routine practice (“normalisation”) of complex interventions. We used the same approach for the intervention development stage [21]. All analysis was conducted according to the standard procedures of rigorous qualitative analysis [45]. We used procedures from first-generation grounded theory (coding, constant comparison, memoing) [46], from analytic induction (deviant case analysis) [47] and from constructionist grounded theory (mapping) [48]. We undertook independent coding and cross checking, and a proportion of data was analysed collectively in ‘data clinics’ where the research team shared and exchanged interpretations of key issues emerging from the data. Pseudonyms were used for all participant names in the transcripts and in any quotes used.

Descriptive statistics were used to summarise quantitative data on rates of eligibility, consent, recruitment and retention; summary statistics were also included for assessment and outcome measures.

## Results

**Fig. 1:**
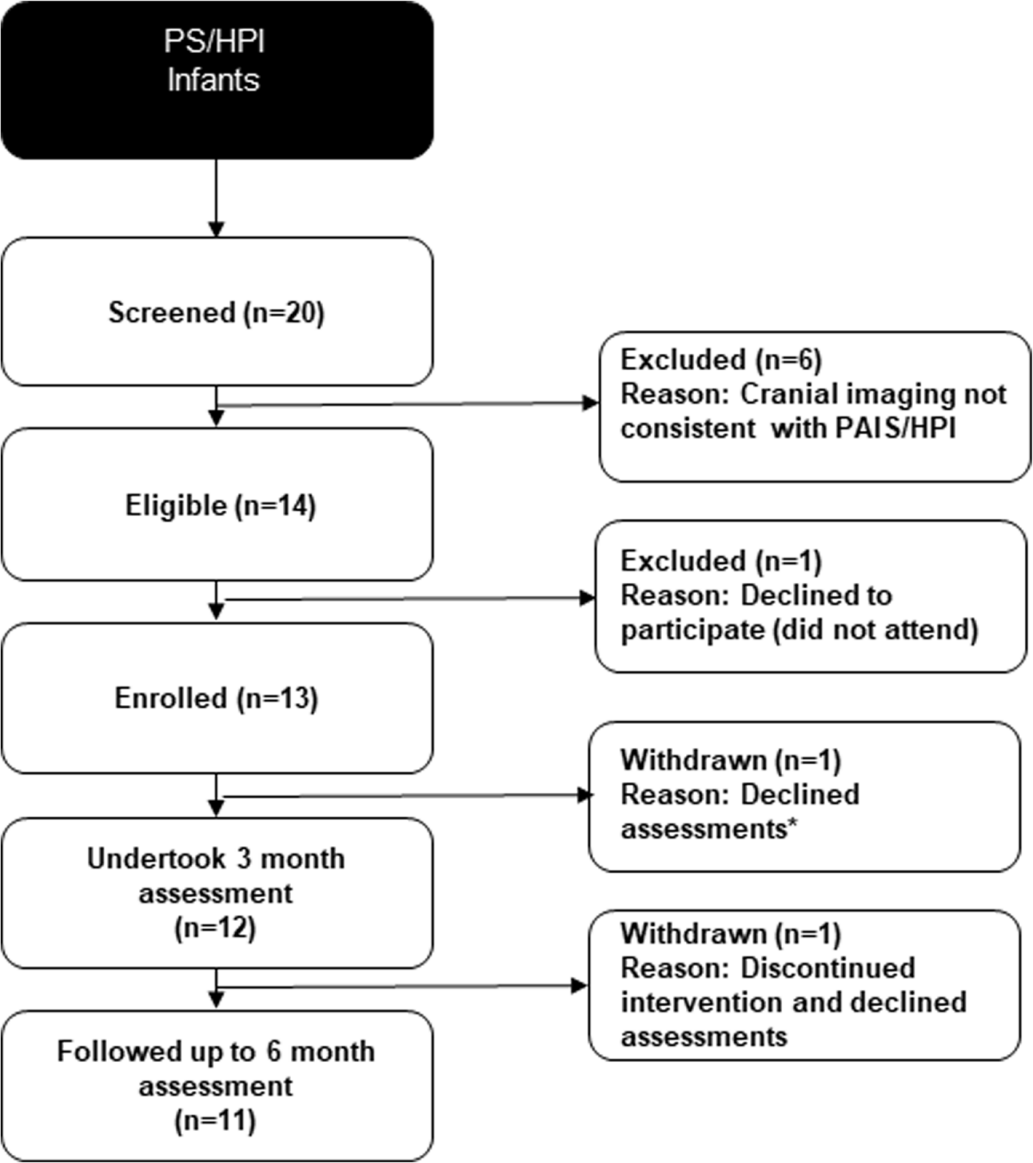
Patient flow (participants with PS/HPI)

Figure 1 shows the flow diagram for participants with PS/HPI. Twenty infants were screened and 14 found to be eligible. Of these, one parent of a preterm infant declined to participate and 13 families were enrolled (6 PS; 7 HPI). Two other parents of preterm infants withdrew from the study, so 11 families were followed up to the 6-month assessment. One parent who withdrew expressed a feeling of being overwhelmed by visits from healthcare professionals and the other felt that participation caused her to dwell excessively on her infant’s medical problems. Interestingly, this latter parent made contact several months later, asking to be re-enrolled in the study to participate in the interview. During the in-depth interview, she commented that she had continued to follow the eTIPS approach after study withdrawal.

We also approached and screened 14 TD infants. One preterm (23-week gestation) TD infant died of a respiratory infection prior to undertaking any assessments. Another TD infant was excluded due to the subsequent identification of a medical condition affecting eligibility; thus, we could analyse data for 12 TD infants to age 6 months.

Participant enrolment started in August 2015 and was completed in January 2017 for infants and carers and for therapists by June 2017.

Table 2 shows the demographic and clinical characteristics for each group. There were 4 missed/cancelled visits in TD infants (three at 5 months and one at 4 months) but none in the infants with HPI and only one missed/cancelled visit in an infant with PS (at 5 months, which included the qualitative interview). This was inevitable due to the personal circumstances of the infant and family at the time.

**Table 2:** Baseline demographic and clinical characteristics for each group

		PS	HPI	TD
Number		6	7	13
Number Term-born		5	0	8
Gestational age for preterm infants (weeks)	Median	35 (*n* = 1)	27	31
	Range	n/a	23–30	24–35
Birthweight (g) for preterm infants	Median	2575 (*n* = 1)	786	1361
	Range		550–1300	740–1644
Number of males		2	6	5
Parents/carers recruited (M, F, GM, GF)		6, 6, 1, 0	7, 5, 0, 1	13, 12, 0, 0
Side of brain lesion (L, R, N/A)		3, 3	4, 3	N/A

*M* mother, *F* father, *GM* grandmother, *GF* grandfather

### Feasibility and acceptability

Table 3 summarises the results of the eTIPS feasibility questionnaire.

**Table 3:** eTIPS Feasibility Questionnaire

Item	Description	Mother	Mother	Father	Father
		1 m	6 m	1 m	6 m
	Number in section A	12	*11*	*9*	*7*
A1	I understand the purpose of eTIPS	5 (4–5)	*5 (4–5)*	*5 (4–5)*	*5 (4–5)*
A2	I understand the types of things eTIPS requires me to do with my child	*5 (4–5)*	*5 (4–5)*	*5 (4–5)*	*5 (4–5)*
A3	I can see the potential value of eTIPS for my child	5 (4–5)	5 (4–5)	5 (4–5)	*5 (4–5)*
A4	I can easily fit eTIPS into my day	5 (3–5)	5 (4–5)	5 (3–5)	4 (3–5)
A5	It is easy to carry out the eTIPS approach with my child	5 (2–5)	5 (4–5)	4 (3–5)	4 (4–5)
A6	Using eTIPS disrupts my relationship with my child	0.5 (1–3)	1 (1–2)	2 (1–3)	1 (1–2)
A7	Sufficient training is provided for me to use eTIPS with my child	4.5 (3–5)	5 (2–5)	5 (3–5)	4 (3–5)
A8	My child tolerates eTIPS well	4.5 (3–5)	4 (4–5)	4 (4–5)	*5 (4–5)*
	Number in section B	11	11	9	7
B1	When you use eTIPS, how familiar does it feel?	6.97 (1.91)	9.14 (0.84)	5.71 (2.44)	7.69 (1.76)
B2	Do you feel eTIPS is currently a normal part of your day/time with your child?	6.95 (1.90)	9.36 (0.81)	6.24 (2.07)	7.81 (2.07)
B3	Do you feel eTIPS will become a normal part of your day/time with your child?	9.13 (1.29)	9.45 (0.82)	9.26 (0.78)	8.97 (0.94)

A 5 point Likert scale was used: 1 = “strongly disagree”; 2 = “disagree”; 3 = “neither agree nor disagree”; 4 = “agree”; 5 = “strongly agree”. Median values for each of items A1–8, with minimum and maximum in brackets. For items B1–3, mean and standard deviation are given as these were represented as a continuous scale (0–10), with increasing scores representing increasing familiarity with/perceived normality of the approach

Eleven families with an infant with PS/HPI took part in in-depth interviews (six with only the mother; five with both parents), as did thirteen families with a TD infant (twelve with only the mother, one with both parents), and six therapists, who between them were supporting ten of the infants with PS/HPI. From the interviews, we gained a number of insights. Firstly, parents were very willing to enter the study to make sure that they had done everything they could to help their child’s future:

like it took us like five minutes to decide ‘cause I was like, “If we don’t do it and he is left with obvious like damage then we, we would always think, ‘What if we’d done that, that thing and it might’ve made it better?’” (Helen).

In addition to this they felt that there was a very low risk of harm to and that ‘this is less invasive, this trial’ (Barney), in comparison to others they were offered at the time as well as seeming to hold face validity:

and I know it isn't backed up yet 'cause it is a trial, but this feels more like, er, “Surely this has got to work”. It feels like there's more science behind it (Barbara).

The high-quality materials were appreciated by the families: ‘It’s not a cheap piece of paper or a cheap, you know, stapled sheets of paper together, it’s a full-on book’ (Selina), as was the layout and format. Although at first they reported that the size of the manual looked a little daunting, they quickly came to appreciate that the information was simple and easy to follow:

It wasn't really too bad once you start looking through the book you… At first, yes, it does sound like a hell of a lot. Once you start looking through the book, it's sort of like really easy to integrate into day-to-day things that you do with, with the child (Belinda).

The eTIPS approach integrated into family life relatively easily, particularly when parents had been given information before leaving the hospital. It quickly became a normal and pervasive part of their everyday interactions with their child:

It is second nature now as we did it this way from coming home from hospital, it's routine, we don’t have to think about it (Emma).

Parents of TD infants were comfortable with the use of infant massage; some were also attending baby massage classes independently.

As well as learning from the eTIPS materials, parents also modelled researcher behaviours in interactions with their children. In particular, parents were often inspired by observing the Hand Assessment for Infants and sourced similar toys to those used in the assessment. Feedback from the team regarding the HAI assessment helped parents to understand how to focus on specific developmentally appropriate areas of practice with their infant. Parents were also appreciative of the positive reinforcement they received from the eTIPS team and of the positive interactions with their infants during visits. The assessments were also considered acceptable, though one parent felt uncomfortable with the use of accelerometers.

Parents were prepared to alter their behaviours and the environment around their child to fit in with the eTIPS approach. Siblings also became involved:

She [the mother] tells them [siblings] to stand on the side where the yellow rattle is so even the quite young ones can understand that and go and stand on that side and sing to him and things (Researcher observation).

Parents rightly did not feel restricted to the suggestions in the manual for promoting activity of the potentially affected side: there were many examples of parents being resourceful and innovating or generalising the eTIPS approach to fit in with their lives. This included methods they had found to encourage others to approach and interact with their infant from the affected side, e.g. by considering the positioning of their infant’s pram or crib within the environment. They also felt that the eTIPS approach was a positive factor in their interactions with their infants:

I never used to get smiles or anything like that. And I think all the eTIPS and stuff, and the playing, and stuff like that, and the different toys, I think that… And obviously I’ve been getting more smiles. Everyone gets them, apart from me. And I’ll get upset. But I think, like, this has helped him interact with me. (Fiona).

Therapists agreed with the approach and could understand the science/evidence behind it. Therapists reported demonstrating therapy activities and leaving advice for families to work on specific activities between sessions as part of their normal practice. They liked the eTIPS materials and felt they assisted them in teaching the families without having to spend a lot of time pre-preparing personalised therapy plans. They appreciated the pervasiveness of the approach and felt that it would lead to better outcomes.

Yeah, erm, I really don’t think the approach is too different, like I said, to anything that I would do anyway. I suppose it’s just more structured and a lot more information for parents. But, that’s what you want. We go in once a week maximum. It’s no good us just doing something with them once a week. You know, it needs to be done all the time. (Nicola).

The idea of integrating the therapy into daily activities as opposed to being a separate ‘therapy session’ was also seen as useful by therapists:

'Cause, what I always say to any parent, is when I give them their programme, I always put, “Integrate this into your daily play. Don’t make it, right, now we’re doing your physio.” (Nicola).

The regular communication between the eTIPS team and families was acceptable to all. There was variation in how families responded to text messages and phone calls. Some replied regularly and gave the team updates about their infant, while some families rarely responded but when questioned did not want the messages to stop. They appreciated that the team were available for specific questions and support.

I like the texts as well, but, er, I, I like that fact that you just keep in touch consistently, because then… And I’ve got all the numbers as well, so if anything happens I know that you’re just at the end of the phone. (Deirdre).

Maintaining this communication also reinforced the importance of eTIPS, their involvement in it and the research team’s commitment:

It [text messages and phone calls] makes us feel, me and Samuel, and, and dad, important that, you know, again it isn’t just a paper exercise….that we have actually got full involvement in something which is hopefully going to be making a difference. (Selina).

Infants sustained no adverse events related to the eTIPS intervention: no injuries were reported from the activities undertaken and no infant developed a preference for the hand contralateral to the side of the brain affected by the lesion.

### Assessments

It was possible to undertake the required assessments within the context of a 1 h visit, fitting them around the infant’s needs and parental requests for information and support.

**Fig. 2:**
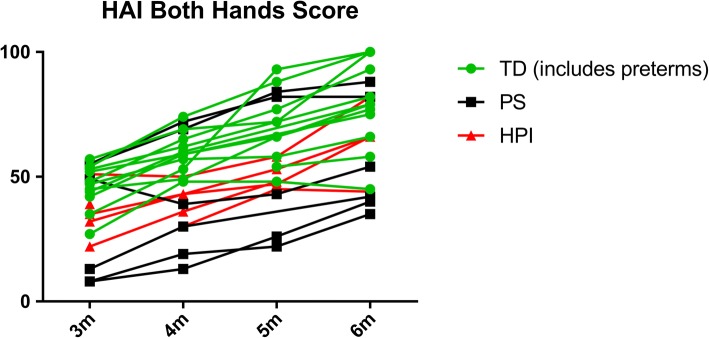
Hand Assessment for Infants

Figure 2 shows the individual 0–100 HAI scores for the group. For the 11 affected infants with HAI data at 6 months, the mean Both Hands Score was 58.5 HAI units (s.d. 19.1). Change scores for the HAI from 3 to 6 months were available for 12 TD and 10 affected infants. For TD infants, the mean change score was 32.4 HAI units (s.d. 17.1 units; 95% CI 21.5 to 43.3). For infants with stroke the mean change score was 27.2 HAI units (s.d. 8.5 units; 95% CI 21.1 to 33.3). At age 6 months the mean asymmetry index in the affected group was 35.5% (95% CI 12.8–58.1); for TD infants, it was 5.2% (95% CI -2.0 to 12.3). Table 4 summarises findings in relation to the other assessments piloted. In addition, multiple improvements to the healthcare resource use data collection forms were made in response to feedback and evidence of need for improved clarity. Table 5 summarises the neuroimaging findings in relation to the HAI Both Hands scores at 6 months.

**Table 4:** Summary of findings from other assessments piloted

Assessment	Findings	Implications for future trial
HAI	Assessments generally enjoyed by infants and perceived as valuable by parents in demonstrating their infant’s abilities, identifying challenges to work on and modelling strategies.	Valuable assessment, worth the training required for therapists to undertake and score. Resource implications: need to video and upload assessments for later scoring.
PSOM	Useful clinical proforma though in the context of the other data collected (HAI, GMs and AIMS), the motor summary scores were not required, and the cognitive, behavioural and language scores were more suited to older infants.	Useful for summarising longer term outcomes and for comparison with other infants with PS/HPI. The HINE would be another option.
GM	Straightforward to undertake, video record and score. Two infants showed fidgety movements (predictive of good motor outcome) by 4 m which were not seen at 3 m.	Provides early indicator of likely normal vs. abnormal motor outcome. For centralised scoring, video upload to a central server is required.
Accelerometry	Time-consuming and at times technically challenging; one parent uncomfortable with use. Analysis complex.	Valuable exploratory data but current approach unsuitable for RCT given resources required.
AIMS	Easy to obtain and score. AIMS at 6 m were 25th centile or above for all except one TD term infant (10–25 centile) but lower for preterm TD and PS/HPI infants (one exception with small cortical infarct and good outcome).	Useful to describe early gross motor function which impacts hand use. However, abnormal motor patterns seen in infants with evolving neurology could distort scores.
WEBWMS	All returned questionnaires were fully completed. Two mothers of TD infants at baseline and two at 6 m failed to return questionnaires. Questionnaires from fathers were less frequently returned (3 TD missing at start and end; 3 PS/HPI missing at end). Change scores did not suggest any adverse effect of eTIPS on parental mental wellbeing: PS/HPI maternal change score 2.2 (95% CI -3.9 to 8.3; *n* = 10). TD maternal change score − 3.2 (95% CI -9.4 to 3.0, *n* = 10); higher scores represent better mental wellbeing.	Questionnaire return rate optimised by sending out forms prior to visit, bringing spare forms and collecting them during the visit. Extra vigilance required to obtain questionnaires from fathers.
PSOC	Questionnaire return rate same as WEBWMS but multiple non-completed items which qualitative data suggested were due to reluctance to answer questions perceived as sensitive, as well as initial failure of some fathers to complete the reverse of the form.	An alternative and positively framed questionnaire addressing aspects of parental sense of competence could be used, e.g. Family Empowerment Scale.

**Table 5:** Imaging findings and HAI Both Hands scores at 6 months

No.	Imaging	Side (brain)	Lesion type	Description	6 m HAI Both Hands
1	CrUSS, MRI	Right	Infarct	Right cerebral cortex & PLIC; left occipital lobe infarct	35
2	MRI	Right	Infarct	MCA territory infarct involving cortex, PLIC & corticospinal tracts	42
3	MRI	Left	Infarct	Left frontoparietal; small left posterior parietal & tiny right frontal subcortical lesion	88
4	CT, MRI	Left	Infarct	Segmental MCA territory infarct involving frontal & parietal lobes.	54
5	CrUSS, MRI	Left	Infarct	Anterior circulation infarct affecting cortical & subcortical structures	82
6	CT, MRI	Right	Infarct, SAH, IVH	Extensive MCA territory infarct involving cortical & subcortical structures, basal ganglia & corticospinal tract	40
7	CrUSS	Right	HPI	Frontal lobe	45
8	CrUSS	Left	HPI	Frontoparietal	n/a
9	CrUSS	Right	HPI	Adjacent to body of lateral ventricle	66
10	CrUSS	Right	HPI	Adjacent to body of lateral ventricle, extending to temporal lobe	44
11	CrUSS	Left	HPI	Left periventricular	66
12	CrUSS	Left	HPI	Left frontoparietal	n/a
13	CrUSS	Left	HPI	Frontotemporal	82

*CrUSS* Cranial Ultrasound, *PLIC* Posterior limb of internal capsule, *MCA* middle cerebral artery

## Discussion

The eTIPS intervention was, in general, extremely well received and appreciated by families, and fitted into their everyday lives. This is likely to be due in part to the involvement of parents and therapists throughout the intervention development process and the use of Normalisation Process Theory [21]. It was important to ensure that parents felt capable of delivering the intervention and were not overburdened or stressed by it; our results support this finding. A supportive, problem-solving approach from the eTIPS team is likely to have influenced this positive outcome: parent-delivered therapy is underpinned by therapists who empower, motivate and support families to deliver effective interventions (Lord et al., under submission).

Therapists were also supportive of the eTIPS approach; this is important in terms of future implementation of the approach. An issue relevant to our planned randomised controlled trial is the need to train therapists at remote sites on the eTIPS approach and assess the fidelity of their delivery of parental training and supervision. Commitment of local teams to the endeavour will be important and we plan to provide centralised support as well as training. We are currently developing a training package, which will itself be piloted prior to use. Central video-based review of selected sessions can be a useful method for assessment of intervention fidelity.

We found the HAI to be a very valuable assessment. Parents were engaged with the assessment and infants enjoyed taking part; researchers could use the HAI to help parents to know what developmental skills to focus on next and how to help the child to develop those skills. It was clear that for parents, the changes in hand function seen from visit to visit were meaningful and important. The HAI gives a detailed summary of hand function not currently available in this age group through any other measure.

Regarding our inclusion criteria, we had hoped to include some infants with presumed perinatal stroke (who typically present with emerging signs of motor problems after the first months of life), by allowing infants aged up to 3 months to enter the trial. However, we did not recruit any such infants, presumably because they had not come to medical attention by this point. Therefore, for a future randomised trial it would be appropriate to restrict recruitment age to 1 month corrected or less, to maximise and standardise the duration of subsequent intervention. We also recruited infants with varying degrees of brain injury, and this also has relevance to our future recruitment strategy. Of the infants with infarcts, the two infants with only cortical and subcortical lesions had excellent motor outcomes, though one of these infants had a 13% difference in hand function on the HAI score which was clinically noticeable. Similarly, infants with extensive lesions also involving the basal ganglia and corticospinal tract had more marked motor involvement in our study. Given the likelihood of a good motor outcome in infants with radiological sparing of the basal ganglia and corticospinal tract and conversely the high likelihood of hemiparesis with involvement of these structures in addition to a cortical/subcortical lesion [5], we will aim in future to include only infants with infarcts predicted to have a moderate or high risk of an abnormal motor outcome. Prompt centralised reporting of imaging findings will be necessary to ensure this. We also intend to explore the relationship between imaging findings and outcome as part of a mechanistic evaluation.

The influence of initial radiological findings on motor outcome in infants with HPI was less clear based on cranial ultrasound, as predicted from the literature [11, 12]; imaging with MRI in this latter group is not part of standard practice and would need to be incorporated into a trial protocol. Importantly, the existence of a relationship between initial imaging findings and motor outcome should not preclude attempts to improve outcome through intervention.

The retention rate in our trial was high. A few mothers of preterm infants struggled with the overall burden of care for those infants due to other morbidity related to their prematurity, making it harder for them to take on additional commitments related to the research. Based on their feedback we would see this as reflecting an increased need for support of these parents rather than a reason to exclude preterm infants from the intervention per se. With preterm infants, there may be an advantage in starting parent training in eTIPS before the infant is discharged from hospital, to offset some of this burden. Overall, parents in both the PS and HPI groups felt that their infants benefitted from the approach. Whilst one parent of a preterm infant disengaged from eTIPS assessments because she felt she was dwelling excessively on her infant’s medical problems as a result, she continued to deliver the eTIPS approach. This highlights a challenge faced by researchers in delivering an intervention such as eTIPS: parents need to understand the rationale for the intervention, which includes an awareness that their infant is at risk of developing a motor disability which the intervention aims to mitigate against. Assessments, whilst essential, may augment anxiety in parents as they seek to determine whether any signs of motor disability have emerged. This is a strong argument for rationalising the assessment profile to key time points in the planned future definitive trial, for encouraging parents to see the progress their infants have made and for providing emotional support.

The assessments piloted were in the most part suitable for use in a large-scale trial. The main exception to this was the PSOC for which parents often omitted to provide answers for certain items. Findings from qualitative data analysis indicated that parents found some of the questions sensitive and were wary of providing written answers. We included the questionnaire because we wanted to be able to demonstrate in a trial that parenting confidence was not adversely affected by participation. However, our qualitative data findings indicate that parents generally found participation a positive experience and the WEBWMS suggested that parental wellbeing did not decline. One option would be to include a parental empowerment scale such as the Psychological Empowerment Scale [49] (though this frames questions to parents as if they have already acknowledged their child to have a disability, which is inappropriate for parents of such young infants) or the Family Empowerment Scale [50], though another (preferred) option would be simply to omit the PSOC without replacement. Similarly, although the Pediatric Stroke Outcome Measure (PSOM) allows comparison of motor outcomes to those of other infants with PS, the Hammersmith Infant Neurological Examination (HINE) would be a useful addition given its psychometric properties [51].

To progress to a randomised controlled trial one further issue to be addressed was that of randomisation. As we needed to maximise feedback regarding the trial materials, we did not randomise infants in the feasibility trial. However, since completing the trial we conducted a workshop with 8 parents (4 couples) who took part, to obtain their views on randomisation. Parents felt strongly that their infants had benefited from the eTIPS approach; through discussion they understood that a randomised trial would be necessary to provide definitive evidence regarding benefit. Knowing that infants in the standard care arm of the trial would still receive regular therapist review meant that all parents agreed that eTIPS should proceed to a randomised controlled trial.

Conducting a randomised controlled trial of a behaviour change intervention will have its challenges, not least in terms of avoidance of contamination, for which we have plans in place [52]. Issues such as competitive therapy bias have affected previous therapy trials by reducing the difference between the interventions provided to the intervention and control group [53]. Cluster randomisation is one way to avoid such problems, but has the disadvantage of introducing potential bias due to differences in other aspects of care between clusters. Another option is to have a group of trial therapists who oversee the intervention and a separate group who oversee standard care. Many UK sites will be required, and therapists may be employed by different organisations (and therefore sites) from the recruiting clinicians for any one infant: the trial will require expert input from a clinical trials manager to assist with these issues. However, our positive experiences regarding this feasibility trial indicate that we should proceed.

## Conclusions

The eTIPS intervention was feasible to deliver and acceptable to families and therapists. We plan to investigate efficacy of this parent-delivered early intervention in a multicentre randomised controlled trial.

## Abbreviations

AIMS: Alberta infant motor scale
CIMT: Constraint-induced movement therapy
CrUSS: Cranial ultrasound
eTIPS: Early therapy in perinatal stroke
GM: General movements
HAI: Hand assessment for infants
HINE: Hammersmith infant neurological examination
HPI: Haemorrhagic parenchymal infarction
MCA: middle cerebral artery
PLIC: Posterior limb of internal capsule
PS: Perinatal stroke
PSOC: Parenting sense of competence scale
PSOM: Pediatric stroke outcome measure
TD: Typically developing
UCP: Unilateral cerebral palsy
WEBWMS: Warwick-Edinburgh Mental Wellbeing Scale
